# Patterns of chlamydia testing in different settings and implications for wider STI diagnosis and care: a probability sample survey of the British population

**DOI:** 10.1136/sextrans-2016-052719

**Published:** 2016-12-15

**Authors:** Soazig Clifton, Catherine H Mercer, Sarah C Woodhall, Pam Sonnenberg, Nigel Field, Le Lu, Anne M Johnson, Jackie A Cassell

**Affiliations:** 1 Centre for Sexual Health and HIV Research, UCL, London, UK; 2 HIV & STI Department, National Infection Service, Public Health England, London, UK; 3 Division of Primary Care and Public Health, Brighton and Sussex Medical School, Brighton, UK

**Keywords:** CHLAMYDIA TRACHOMATIS, SCREENING, HIV TESTING, GENERAL PRACTICE

## Abstract

**Background:**

Following widespread rollout of chlamydia testing to non-specialist and community settings in the UK, many individuals receive a chlamydia test without being offered comprehensive STI and HIV testing. We assess sexual behaviour among testers in different settings with a view to understanding their need for other STI diagnostic services.

**Methods:**

A probability sample survey of the British population undertaken 2010–2012 (the third National Survey of Sexual Attitudes and Lifestyles). We analysed weighted data on chlamydia testing (past year), including location of most recent test, and diagnoses (past 5 years) from individuals aged 16–44 years reporting at least one sexual partner in the past year (4992 women, 3406 men).

**Results:**

Of the 26.8% (95% CI 25.4% to 28.2%) of women and 16.7% (15.5% to 18.1%) of men reporting a chlamydia test in the past year, 28.4% of women and 41.2% of men had tested in genitourinary medicine (GUM), 41.1% and 20.7% of women and men respectively tested in general practice (GP) and the remainder tested in other non-GUM settings. Women tested outside GUM were more likely to be older, in a relationship and to live in rural areas. Individuals tested outside GUM reported fewer risk behaviours; nevertheless, 11.0% (8.6% to 14.1%) of women and 6.8% (3.9% to 11.6%) of men tested in GP and 13.2% (10.2% to 16.8%) and 9.6% (6.5% to 13.8%) of women and men tested in other non-GUM settings reported ‘unsafe sex’, defined as two or more partners and no condom use with any partner in the past year. Individuals treated for chlamydia outside GUM in the past 5 years were less likely to report an HIV test in that time frame (women: 54.5% (42.7% to 65.7%) vs 74.1% (65.9% to 80.9%) in GUM; men: 23.9% (12.7% to 40.5%) vs 65.8% (56.2% to 74.3%)).

**Conclusions:**

Most chlamydia testing occurred in non-GUM settings, among populations reporting fewer risk behaviours. However, there is a need to provide pathways to comprehensive STI care to the sizeable minority at higher risk.

## Introduction

Genital chlamydia infection is the most commonly diagnosed STI in Britain, with >200 000 diagnoses made in England in 2015,^1^ and an estimated population prevalence of 1.5% in women and 1.1% in men aged 16–44 years.^2^ Chlamydia is easily treated with antibiotics; however, infection is usually asymptomatic and therefore can often go undiagnosed and untreated, which may lead to serious adverse sequelae including pelvic inflammatory disease, ectopic pregnancy and tubal infertility.^3^ Since the early 2000s, chlamydia testing has been expanded in all British countries in line with national sexual health strategies with the aim of increasing testing among those aged under 25 years who are sexually active, the group most affected by chlamydia.^4–6^ A National Chlamydia Screening Programme (NCSP) was established in England in 2003, which recommends opportunistic screening of men and women aged under 25 years annually and on change of sexual partner in a range of healthcare and non-healthcare settings.^7^ In Scotland and Wales, testing of asymptomatic young adults, particularly those at higher risk, is recommended although there is no formal screening programme.^6^ ^8^ ^9^

Partner notification and testing for other STIs including HIV are recommended for individuals diagnosed with chlamydia regardless of the setting. This may require referral to genitourinary medicine (GUM), where the treatment service is unable to provide these services.^10–12^ However, while GUM services routinely undertake a sexual history and assess risk of other STIs, this is not an established standard of care for all those testing for chlamydia in general practice (GP), where indeed many health practitioners are not trained to make such an assessment. Therefore, although the expansion of chlamydia testing settings has the potential to reach those who would not usually attend GUM services, there could be a missed opportunity for diagnosis of other STIs if high-risk individuals test in these settings.

Prior to the NCSP, coverage of chlamydia testing in England within GP was low, with women aged ≥25 years being disproportionately tested, and very few tests carried out in men.^13^ ^14^ Analysis from a representative population survey—the second National Survey of Sexual Attitudes and Lifestyles (Natsal-2), undertaken in 1999–2001, before the NCSP was introduced—found that those diagnosed with chlamydia in GP reported lower risk behaviours than those diagnosed in GUM.^15^ More recent NCSP and Natsal data show that positive chlamydia tests are more common in GUM than GP or other settings,^1^ ^16^ suggesting that those tested outside GUM are still lower risk; however, up-to-date data on the demographic and behavioural characteristics of those testing in different settings are needed. In this paper, we compare characteristics of those aged 16–44 years tested for chlamydia in GUM, GP and other settings using data from Natsal-3, a British probability sample survey undertaken 2010–2012.

## Methods

### Participants and procedures

Natsal-3 was a stratified probability sample survey of 15 162 men and women aged 16–74 years in Britain, interviewed in 2010–2012. The overall response rate was 57.7% (of all known or estimated eligible addresses). Participants were interviewed using a combination of computer-assisted face-to-face and self-completion questionnaires. Non-response to individual questions (missing data) was low, typically 1–3%. Full details of the methods used in Natsal-3 have been reported elsewhere.^17^ ^18^

Here, we present results for 8397 participants (4992 women, 3405 men) aged 16–44 years with at least one sexual partner in the past year, and further analysis of 2349 participants (1610 women, 739 men) who also reported being tested for chlamydia in the past year.

### Statistical analysis

All analyses were carried out using Stata V.13, (Stata Statistical Software: Release 13. College Station, Texas, USA: StatCorp, 2013) accounting for stratification, clustering and weighting of the sample. Data were weighted to account for differential selection to the survey and to address non-response bias by age, sex and region. We present demographic and behavioural characteristics (percentages and 95% CIs) of those tested for CT in the past year by most recent testing location: sexual health (GUM) clinic, GP surgery (GP) or ‘other non-GUM settings’ (comprising National Health Service (NHS) family planning clinic; school, college or university; antenatal service; termination of pregnancy clinic; private (non-NHS) clinic; hospital accident and emergency; pharmacy; youth advisory clinic; internet and other non-healthcare setting). We used multinomial regression to calculate relative risk ratios (RRRs), comparing the demographic and behavioural characteristics of those tested in GP or other locations with those tested in GUM. For each comparison (eg, ‘other non-GUM settings’ vs GUM), the RRRs correspond to the ORs ignoring the third location category (eg, GP). These analyses were initially stratified by broad age group (16–24/25–44 years) where numbers permitted but as the pattern of associations was similar for both groups, final analyses present unadjusted data for all aged 16–44 years combined, with age-adjusted RRRs (aRRRs) presented in the text where adjustment affected associations. We report HIV testing among those diagnosed with chlamydia, by location of chlamydia treatment, with the time frame expanded to the past 5 years due to small numbers diagnosed with chlamydia in the past year.

## Results

Of all participants aged 16–44 years with at least one sexual partner in the past year, 26.8% (95% CI 25.4% to 28.2%) of women and 16.7% (15.5% to 18.1%) of men reported a chlamydia test in the past year. Testing was more common in those aged 16–24 years (55.4% (52.6% to 58.2%) of women, 36.0% (33.2% to 38.9%) of men), the age group eligible for the NCSP in England (table 1). Across all aged 16–44 years who had been tested, 28.4% (25.8% to 31.9%) of women and 41.2% (37.5% to 45.9%) of men had been tested in GUM, 41.1% (38.4% to 44.0%) and 20.7% (17.6% to 24.2%) of women and men respectively had been tested in GP, and 30.5% (28.0% to 33.1%) and 37.7% (33.6% to 41.9%) of women and men respectively had been tested in other non-GUM settings including NHS family planning clinic, antenatal service, termination of pregnancy clinic, school, college, university—henceforth referred to as ‘other non-GUM settings’. Internet-based testing was rare, reported by only 0.6% (0.3% to 1.1%) of women and 1.9% (1.1% to 3.2%) of men tested for chlamydia in the past year. Younger people were more likely to report internet-based testing, reported as the location by 1.7% (1.0% to 2.6%) of people aged 16–24 years compared with 0.4% (0.2% to 1.0%) of people aged 25–34 years, and noone aged 35–44 years.

**Table 1:** Chlamydia testing in the past year and location of most recent test, by age and sex

	Women (years)								Men (years)							
	16–24		25–34		35–44		16–44		16–24		25–34		35–44		16–44	
	%	95% CI	%	95% CI	%	95% CI	%	95% CI	%	95% CI	%	95% CI	%	95% CI	%	95% CI
Tested in the past year	55.4	52.6–58.2	23.7	21.7–25.8	9.2	7.6–11.1	26.8	25.4–28.2	36.0	33.2–38.9	15.1	13.1–17.3	4.3	3.1–6.0	16.7	15.5–18.1
*Denominator (unwt, wt)*	*(1680, 932)*		*(2242, 1250)*		*(1059, 1301)*		*(4981, 3483)*		*(1298, 948)*		*(1377, 1239)*		*(723, 1304)*		*(3398, 3491)*	
Testing location, among those tested																
Sexual health (GUM) clinic	29.2	25.7–32.9	30.4	25.7–32.9	20.4	13.5–29.6	28.4	25.8–31.9	31.0	26.3–36.1	60.0	52.8–66.7	45.7	29.8–62.6	41.2	37.5–45.9
GP	35.0	31.6–38.5	35.0	31.6–38.5	55.6	56.7–65.2	41.1	38.4–44.0	17.1	13.8–21.1	22.2	16.8–26.7	37.5	22.7–55.1	20.7	17.6–24.2
NHS family planning clinic	9.1	7.3–11.3	9.1	7.3–11.3	8.9	4.5–16.9	8.9	7.5–10.6	4.4	2.7–6.9	3.7	1.8–7.3	4.6	1.0–18.7	4.2	2.9–6.0
School, college, university	11.6	9.4–14.1	0.9	0.4–2.1	0.0	–	6.7	5.5–8.2	24.2	20.0–28.8	1.7	0.6–4.5	1.7	0.2–11.1	14.8	12.1–17.9
Antenatal service	3.0	2.1–4.3	3.0	2.1–4.3	5.9	2.7–12.4	4.5	3.5–5.7	n/a	n/a	n/a	n/a	n/a	n/a	n/a	n/a
Termination of pregnancy clinic	0.4	0.2–1.0	0.4	0.2–1.0	1.9	0.4–7.3	0.9	0.5–1.5	n/a	n/a	n/a	n/a	n/a	n/a	n/a	n/a
Other healthcare settings*	1.9	1.2–3.1	1.9	1.2–3.1	6.1	2.5–14.1	2.6	1.8–3.7	1.6	0.8–3.3	2.5	1.0–5.7	1.3	0.2–9.0	1.8	1.1–3.1
Other non-healthcare settings†	5.7	4.3–7.6	5.7	4.3–7.6	1.2	0.2–7.7	3.8	2.9–4.9	16.0	12.2–20.6	2.8	1.5–5.2	0.0	–	10.2	7.9–13.2
Somewhere else	4.1	2.9–5.9	4.1	2.9–5.9	0.0	–	3.1	2.3–4.2	5.9	4.0–8.7	7.3	4.0–13.2	9.2	3.0–24.7	6.7	4.8–9.2
*Denominator (unwt, wt)*	*(933, 516)*		*(565, 296)*		*(112, 120)*		*(1610, 933)*		*(468, 341)*		*(230, 187)*		*(41, 56)*		*(739, 584)*	

Denominator for percentage tested in the past year is those aged 16–44 years reporting at least one sexual partner in the past year. Denominator for testing location is (of these) those reporting a test in the past year.

*Comprises private (non-NHS) clinics or doctor, Hospital Accident and Emergency department, pharmacy/chemist.

†Comprises youth advisory clinic (eg, Brook clinic), internet, other non-healthcare place (eg, youth club, festival, bar).

GP, general practice; GUM, genitourinary medicine; NHS, National Health Service; unwt, unweighted denominator; wt, weighted denominators.

Women who most recently tested for chlamydia in GP were more likely to be older, living with a partner, have no educational qualifications and living in rural areas than those tested in GUM (table 2). The association with educational qualifications became non-significant after adjustment for age (aRRR=0.66 (95% CI 0.41 to 1.07); p=0.09). Women tested in other non-GUM settings were more likely to be aged 25–34 years, living with a partner, a student in full-time education and to live in rural areas compared with those tested in GUM. There were no demographic differences between men who had tested for chlamydia in GP and those who tested at GUM clinics (table 3). However, men tested for chlamydia in other non-GUM settings were younger, less likely to be living with a partner and more likely to be students compared with those tested in GUM; these associations remained after adjustment for age (data not shown).

**Table 2:** Demographic characteristics of those tested for chlamydia in the past year, by location of test—women

	Characteristics of women reporting a chlamydia test in the past year						Multinomial regression analysis			
	Tested in sexual health (GUM) clinic		Tested in GP		Tested in other settings		Tested in GP vs GUM		Tested in other settings vs GUM	
	%	95% CI	%	95% CI	%	95% CI	RRR	95% CI)	RRR	95% CI
*Women*										
Age (years)										
16–24	56.8	50.7 –62.6	47.1	42.8–51.5	65.2	60.2–70.0	1.00	–	1.00	–
25–34	34.0	28.6–39.8	35.5	31.5–39.7	24.6	20.8–28.9	1.26	0.93–1.71	0.63	0.45–0.89
35–44	9.2	6.1–13.8	17.4	13.6–21.9	10.1	6.9–14.7	2.27	1.32–3.92	0.96	0.50–1.83
Country of residence										
England	90.5	87.1–93.1	87.0	83.8–89.6	92.5	90.0–94.4	1.00		1.00	
Scotland	5.3	3.3–8.3	8.3	6.2–11.1	4.4	2.9–6.7	1.63	0.89–2.98	0.81	0.43–1.53
Wales	4.2	2.7–6.4	4.7	3.2–6.8	3.1	2.1–4.6	1.17	0.63–2.18	0.73	0.41–1.28
Relationship status										
Live with a partner (including married)	28.4	23.4–34.1	46.3	41.9– 50.8	37.9	32.9–43.1	1.00	–	1.00	–
In a steady ongoing relationship	31.3	26.4–36.6	29.8	25.9–34.0	33.8	29.4–38.5	0.58	0.41–0.84	0.81	0.55–1.20
Not in a steady relationship	40.3	34.9–46.0	23.9	20.5–27.8	28.3	24.0–33.0	0.36	0.25–0.53	0.53	0.36 –0.77
Education level*										
No academic qualifications	7.5	5.2–10.6	11.4	8.8–14.6	7.1	4.9–10.2	1.00	–	1.00	–
Academic qualifications typically gained at age 16	28.8	23.8–34.3	33.7	29.6–38.0	27.6	23.4–32.2	0.83	0.49– 1.40	1.00	0.55–1.83
Studying for/attained further academic qualifications	63.7	58.1–69.1	54.9	50.5–59.3	65.3	60.4–69.9	0.61	0.38–0.98	1.07	0.61–1.88)
Student in full-time education										
No	79.40	73.8–84.1	83.3	79.8–86.2	70.3	65.5–74.6	1.00	–	1.00	–
Yes	20.6	15.9–26.2	16.7	13.8–20.2	29.7	25.4–34.5	0.78	0.53–1.14	1.63	1.12–2.39
Area of residence										
Urban	89.7	86.5–92.2	83.9	80.1–87.0	84.1	80.0–87.5	1.00	–	1.00	–
Rural	10.3	7.8–13.5	16.1	13.0–19.9	15.9	12.5–20.0	1.68	1.14–2.47	1.65	1.10–2.46
*Denominator (unwt, wt)*†	*457, 265*		*650, 384*		*503, 284*					

Denominator is those aged 16–44 years reporting at least one partner in the past year.

*Participants coded as per their highest academic attainment. Excludes those aged <17 years or with foreign qualifications.

†Denominator is smaller for some analyses due to missing data: 3 women had missing data for relationship status; 60 women had missing education level.

GP, general practice; GUM, genitourinary medicine; RRR, unadjusted relative risk ratio for testing in GP/other settings, compared with testing in GUM; unwt, unweighted denominator; wt, weighted denominator.

**Table 3:** Demographic characteristics of those tested for chlamydia in the past year, by location of test—men

	Characteristics of men reporting a chlamydia test in the past year						Multinomial regression analysis			
	Tested in sexual health (GUM) clinic		Tested in GP		Tested in other settings		Tested in GP vs GUM		Tested in other settings vs GUM	
%	95% CI	%	95% CI	%	95% CI	RRR	95% CI	RRR	95% CI	
*Men*										
Age (years)										
16–24	43.5	37.3– 49.8	48.3	39.5–57.3	80.6	74.5–85.5	1.00	–	1.00	–
25–34	46.0	39.7–52.6	34.3	26.3–43.3	15.2	11.0–20.6	0.67	0.42–1.06	0.18	0.11–0.28
35–44	10.5	6.5–16.6	17.4	10.3–27.7	4.3	2.0–8.7	1.48	0.65–3.38	0.22	0.09–0.56
Country of residence										
England	89.0	84.4–92.4	91.7	85.0–95.6	95.8	88.7–98.5	1.00	–	1.00	–
Scotland	7.7	5.0–11.6	5.4	2.3–11.9	3.40	1.0–11.0	0.68	0.25–1.88	0.41	0.11–1.54
Wales	3.3	1.6–6.7	2.9	1.1–7.2	0.90	0.2–3.9	0.85	0.29–2.52	0.24	0.04–1.42
Relationship status										
Live with a partner (including married)	32.7	26.4–39.7	33.2	24.9–42.8	14.2	10.0–19.8	1.00	–	1.00	–
In a steady ongoing relationship	25.8	20.8–31.6	31.9	23.9–41.1	40.6	34.0–47.6	1.22	0.68–2.18	3.62	2.08–6.32
Not in a steady relationship	41.4	35.0–48.1	34.9	26.7–44.0	45.2	38.4–52.1	0.83	0.47–1.46	2.51	0.70–0.86
Education level*										
No academic qualifications	10.0	6.5–14.9	13.2	7.8–21.3	7.1	3.8–12.8	1.00	–	1.00	–
Academic qualifications typically gained at age 16	36.3	30.4–42.6	44.1	35.5–53.0	25.3	20.0–31.6	0.92	0.42–2.01	0.98	0.43–2.26
Studying for/attained further academic qualifications	53.7	47.0–60.3	42.7	34.3–51.6	67.6	60.7–73.8	0.60	0.28–1.31	1.77	0.78–4.04
Student in full-time education										
No	85.0	79.4–89.2	86.0	78.8–91.0	57.3	50.2–64.2	1.00	–	1.00	–
Yes	15.0	10.8–20.6	14.0	9.0–21.2	42.7	35.8–49.8	0.92	0.49–1.73	4.21	2.60–6.81
Area of residence										
Urban	86.2	81.1–90.0	83.6	76.4–89.0	85.3	80.4–89.2	1.00	–	1.00	–
Rural	13.8	10.0–18.9	16.4	11.0–23.6	14.7	10.8–19.6	1.22	0.68–2.18	1.07	0.66–1.73
*Denominator (unwt, wt)*†	*295, 243*		*151, 121*		*293, 220*		* *	* *	* *	

Denominator is those aged 16–44 years reporting at least one partner in the past year.

*Participants coded as per their highest academic attainment. Excludes those aged <17 years or with foreign qualifications.

†Denominator is smaller for some analyses due to missing data: 1 man had missing data for relationship status; 17 men had missing education level.

GP, general practice; GUM, genitourinary medicine; RRR, unadjusted relative risk ratio for testing in GP/other settings, compared with testing in GUM; unwt, unweighted denominator; wt, weighted denominator.

Compared with GUM, women tested in GP or other non-GUM settings had lower numbers of sexual partners (past year) and were less likely to report: same-sex partners (past year), new partners from outside the UK and overlapping partnerships (both past 5 years) (table 4); these associations remained after adjusting for age (data not shown). Similarly, compared with men tested in GUM, those tested in GP or other locations had lower partner numbers (past year) and were less likely to report same-sex partners (past year), overlapping partnerships (past 5 years), unsafe sex (past year; GP only) or new partners from outside the UK (past 5 years; other settings only) (table 5); these associations remained after adjusting for age (data not shown). Although those tested in GP or other settings were generally less likely to report STI risk behaviours than those tested in GUM, 11.0% (8.6% to 14.1%) of women tested in GP and 13.2% (10.2% to 16.8%) of women tested in other non-GUM settings reported ‘unsafe sex’, defined as at least two partners in the past year and no condom used with any partner in the past year. The corresponding figures for men were 6.8% (3.9% to 11.6%) of those tested in GP and 9.6% (6.5% to 13.8%) of those tested in other settings. Of the women reporting unsafe sex, 85.1% (75.7% to 91.2%) of those tested in GP and 73.1% (59.8% to 83.2%) of those tested in other settings had not attended a GUM clinic in the past year. Among men, 79.8% (64.6% to 89.5%) of those reporting unsafe sex who were tested for chlamydia in GP or other non-GUM settings (combined due to small numbers) had not attended a GUM clinic in the past year.

**Table 4:** Behavioural characteristics of those tested for chlamydia in the past year, by location of test—women

	Characteristics of women reporting a chlamydia test in the past year						Multinomial regression analysis			
	Tested in sexual health (GUM) clinic		Tested in GP		Tested in other settings		Tested in GP vs GUM		Tested in other settings vs GUM	
	%	95% CI	%	95% CI	%	95% CI	RRR	95% CI	RRR	95% CI
*Women*										
Number of partners*, past year										
1	46.9	41.2–52.7	72.0	67.9–75.8	63.2	58.1–68.0	1.00	–	1.00	–
2	19.0	15.0–23.7	11.4	8.8–14.6	16.4	13.2–20.3	0.39	0.26–0.59	0.64	0.43–0.97
3+	34.1	28.7–39.9	16.6	13.6–20.1	20.3	16.8–24.5	0.32	0.22–0.45	0.44	0.31–0.64
Same-sex partner(s), past year	8.9	6.3–12.5	2.7	1.6–4.3	3.9	2.4–6.2	0.28	0.15–0.53	0.42	0.22–0.77
Unsafe sex†, past year	14.7	11.4–18.7	11.0	8.6–14.1	13.2	10.2–16.8	0.72	0.48–1.08	0.88	0.59–1.32
Concurrency in past year‡	22.3	17.9–27.4	8.5	6.5–10.9	14.9	11.6–19.0	0.32	0.22–0.48	0.61	0.41–0.91
New partners from outside UK§, past five years	17.5	13.4–22.6	8.6	6.5–11.3	10.1	7.5–13.5	0.44	0.29–0.68	0.53	0.34–0.83
A sexual partner was concurrent, past five years (yes/probably)	61.5	55.7–67.0	51.1	46.5–55.8	49.1	44.0–54.2	0.65	0.48–0.88	0.60	0.44–0.83
*Denominator* *(unwt, wt)*¶	*457, 265*		*650, 384*		*503, 284*					

*Opposite and/or same-sex partners.

†Defined as two or more partners in the past year and not used a condom in the past year.

‡Overlap between any of three most recent partners in the past  year.

§Includes partners acquired when abroad and when in the UK.

¶Denominator is smaller for some analyses due to missing data: 10 women had missing data for number of partners, 1 for same-sex partners, 6 for unsafe sex, 139 for concurrency and 51 for partners' concurrency.

GP, general practice; GUM, genitourinary medicine; RRR, unadjusted relative risk ratio for testing in GP/other settings, compared with testing in GUM; unwt, unweighted denominator; wt, weighted denominator.

**Table 5:** Behavioural characteristics of those tested for chlamydia in the past year, by location of test—men

	Characteristics of men reporting a chlamydia test in the past year						Multinomial regression analysis			
	Tested in sexual health (GUM) clinic		Tested in GP		Tested in other settings		Tested in GP vs GUM		Tested in other settings vs GUM	
	%	95% CI	%	95% CI	%	95% CI	RRR	95% CI	RRR	95% CI
*Men*										
Number of partners*, past year										
1	40.1	33.9–46.6	54.1	45.1–62.9	49.8	42.9–56.7	1.00	–	1.00	–
2	18.2	13.5–24.1	11.5	7.2–18.0	25.1	19.4–31.9	0.47	0.24–0.92	1.11	0.66–1.87
3+	41.7	35.3–48.4	34.3	26.5–43.2	25.1	20.1–30.8	0.61	0.38–0.99	0.48	0.32–0.74
Same-sex partner(s), past year	11.6	8.1–16.3	2.3	0.8–6.5	3.2	1.8–5.6	0.18	0.06–0.57	0.25	0.12–0.51
Unsafe sex†, past year	14.0	10.3–18.6	6.8	3.9–11.6	9.6	6.5–13.8	0.45	0.23–0.89	0.65	0.38–1.12
Concurrency in past year‡	29.4	23.1–36.6	18.2	12.3–25.9	19.4	13.8–26.7	0.53	0.30–0.93	0.58	0.34–0.98
New partners from outside UK§, past 5 years	27.3	21.7–33.7	22.1	15.7–30.3	13.8	9.9–18.8	0.76	0.45–1.28	0.43	0.26–0.69
A sexual partner was concurrent, past 5 years (yes/probably)	61.9	55.3–68.0	58.8	49.4–67.6	45.6	38.7–52.7	0.88	0.56–1.40	0.52	0.35–0.76
*Denominator (unwt, wt)*¶	*295, 243*		*151, 121*		*293, 220*					

Denominator is those aged 16–44 years reporting at least one partner in the past year.

*Opposite and/or same-sex partners.

†Defined as two or more partners in the past year and not used a condom in the past year.

‡Overlap between any of three most recent partners in the past year.

§Includes partners acquired when abroad and when in the UK.

¶Denominator is smaller for some analyses due to missing data: 3 men had missing data for number of partners, 4 for unsafe sex, 60 for concurrency and 24 for partners' concurrency.

GP, general practice; GUM, genitourinary medicine; RRR, unadjusted relative risk ratio for testing in GP/other settings, compared with testing in GUM; unwt, unweighted denominator; wt, weighted denominator.

A small proportion of men who tested for chlamydia in GP or other non-GUM settings reported sex with another man in the past 5 years (2.3% (0.8% to 6.5%) in GP, 3.2% (1.8% to 5.6%) in other settings). The small numbers prohibit further detailed analysis of this group; however, of the 21 men who have sex with men (MSM) tested for chlamydia in GP/other settings in the past year, only 4 reported an HIV test while 4 reported attending a sexual health clinic in the same time frame.

A more detailed breakdown of the demographic and behavioural profiles by non-GP and non-GUM testing locations can be found in online supplementary appendix tables 1 and 2. Women tested in antenatal services were generally older, living with a partner and reported one partner in the past year. However, approximately 1 in 20 of these women reported unsafe sex or concurrency in the past year, and 2 in 5 thought a partner had been concurrent in the past 5 years.

10.1136/sextrans-2016-052719.supp1 supplementary tables

Of all participants with at least one sexual partner in the past year, 4.3% (3.7% to 4.9%) (n=285) women and 4.2% (3.5% to 5.0%) (n=178) men reported a diagnosis of chlamydia in the past 5 years. Of these, 58.1% (51.0% to 64.6%) of women and 76.7% (68.7% to 83.2%) of men were (most recently) treated in GUM, 26.5% (20.8% to 33.1%) and 18.4% (12.6% to 26.1%) of women and men respectively were treated in GP and 15.4% (10.9% to 21.4%) and 4.9% (2.3% to 10.2%) were treated elsewhere. Overall, 66.1% (59.1% to 72.4%) of women and 56.0% (47.0% to 64.6%) of men treated for chlamydia in the past 5 years reported an HIV test in the same time frame. HIV testing was less common among those treated for chlamydia in GP/other locations (combined due to small numbers) compared with those treated in GUM: 54.5% (42.7% to 65.7%) vs 74.1% (65.9% to 80.8%) for women (p=0.005), 23.9% (12.7% to 40.5%) vs 65.8% (56.2% to 74.3%) for men (p<0.0001). Among women tested for chlamydia in GP/other settings, around half had tested for HIV as part of a sexual health check and 29% due to pregnancy (see online supplementary appendix table 3). Very few women treated for chlamydia in the past year had tested for HIV due to being advised to do so by a doctor (2.9% of those treated in GUM, 1.0% of those treated in GP/other settings).

## Discussion

The majority of those aged 16–44 years who reported recent testing for chlamydia were tested outside GUM, particularly among women, of whom 41.1% tested in GP and 30.5% tested in other non-GUM settings. Women who were tested in GP or other non-GUM settings were more likely to be older, in a relationship and to live in rural areas than those tested in GUM. Women and men tested in GP or other non-GUM settings generally reported lower traditionally ascribed STI ‘risk behaviours’, including in terms of partner numbers, same-sex partners and overlapping partnerships. However, a sizeable minority of those tested outside GUM did report risk behaviours, including unsafe sex (defined as two or more partners and no condoms used with any partner in the past year), which was reported by 11.0% and 13.2% of women tested in GP and other non-GUM settings, and 6.8% and 9.6% of men tested in GP and other non-GUM settings, respectively. The majority of these individuals had not attended GUM in the same time frame. A minority (approximately 3%) of men who had been tested for chlamydia outside GUM reported same-sex partners in the past 5 years, and most of these individuals had not been tested for HIV or attended a GUM clinic in the past year. Despite recommendations that those diagnosed with chlamydia should be tested for other STIs including HIV,^10^ ^12^ the majority of those treated for chlamydia outside GUM in the past 5 years did not report an HIV test in the same time frame.

Our data confirm, at population level, the increase in chlamydia testing in GP and other non-GUM settings reported from NCSP and elsewhere, from a low base in the early 2000s when few men were tested or diagnosed outside GUM and a high proportion of women tested were over the age of 25.^14^ ^19^ ^20^ The finding that a substantial proportion of chlamydia tests are now carried out in GP and other non-GUM settings is in line with routine data collected for people aged15–24 years by the NCSP in England, with around half of these tests occurring in non-GUM settings in 2015,^1^ and routine GP data showing increases in diagnosis rates in GP settings from 22.8 per 100 000 population in 2000 to 29.3 per 100 000 in 2011.^21^ Our data show that around half of those tested in GP and three-fifths of those tested in other non-GUM settings were within the NCSP target age range (under 25 years). Individuals tested in GP and in other non-GUM settings across the 16–44 age range reported lower risk behaviours than those tested in GUM, which is consistent with data from the NCSP.^1^ However, this does not necessarily imply inappropriate testing, for example, previous analyses of Natsal-3 have shown that a substantial amount of chlamydia infections in women aged 16–44 years were among those with only one partner in the past year,^2^ and testing those aged 16–24 years was generally higher among those at greater risk.^16^ Natsal findings on the use of the internet for sexual health have been discussed in more detail elsewhere.^22^ We found lower levels of internet-based testing than the English NCSP data for a similar period.^23^ Furthermore, provision of eSexual Health has increased in recent years therefore the findings presented here are likely to underestimate the current role of internet testing. The finding of lower rates of HIV testing among those treated for chlamydia in non-GUM settings is in line with data suggesting low HIV testing generally, and among patients diagnosed with chlamydia, in GP.^24^ ^25^ Our data confirm from the service user side that MSM and others with more complex STI-related needs are incompletely served in community settings.^26^

The wider strengths and limitations of Natsal-3 have been reported elsewhere.^17^ Strengths include the use of probability sampling methods to obtain a sample broadly representative of the general population in Britain, and the coverage of all three countries in Britain, alongside detailed behavioural data that are not routinely collected in surveillance of testing. As with all surveys, there was non-response to the interview (response rate 58%); however, the sample was weighted to the age, sex and regional profile of the British population, and after this weighting had been applied the sample was generally comparable to the British population on other demographic characteristics.^17^ Our study relies on self-reported data, which may be subject to recall and reporting biases. We only collected data on location of the most recent chlamydia test so our data may not represent all recent testing if participants tested more than once, although there is evidence that retesting rates were low during the study period.^27^ ^28^ Sample size limited our ability to explore testing location by ethnicity or to look at regional differences, and we cannot therefore comment on the relationship between testing venue and known inequalities in STI incidence,^29^ which may be particularly relevant to HIV testing given the known missed opportunities for diagnosis.^30^ We do not know the time sequences of events; therefore, it is possible that HIV testing was unrelated to the chlamydia diagnosis. Given this was a population survey, the number of individuals recently diagnosed with chlamydia was small; therefore, the number treated with chlamydia in GP or other non-GUM settings was insufficient for a detailed analysis even when we expanded the time frame to the past 5 years.

GP and other non-GUM settings make a growing contribution to chlamydia testing. By including behavioural factors in a population-based sample and comparing risk characteristics between settings, our data can inform the planning and targeting of sexual health services. Individuals testing in GP and other non-GUM settings have on average lower rates of STI risk behaviours and are consequently likely to be at lower risk of other STIs including HIV; therefore, the risk of missed opportunities to diagnose other STIs in these groups is unlikely to outweigh the benefits of greater availability of chlamydia testing. However, a sizeable minority of those tested in non-GUM settings reported recent risk behaviours, indicating potential STI-testing needs beyond chlamydia testing; therefore, it is essential that these settings both ensure appropriate care pathways are available for such individuals and ensure individuals become aware of their additional testing and prevention needs. These may include HIV and other STI testing, and in some cases prophylaxis such as human papillomavirus and hepatitis B vaccination and HIV pre-exposure prophylaxis for higher-risk individuals. HIV testing was used as an indicator of whether patients diagnosed with chlamydia received appropriate wider STI care following this diagnosis, as recommended in national guidelines, including specific guidelines for the management of STIs in GP.^10^ ^12^ Although it is concerning that a lower proportion of those treated for chlamydia in the past 5 years in GP and other non-GUM settings reported an HIV test in that time frame, we do not know whether the participants were offered and declined a test, nor whether they were offered or received tests for other STIs or partner notification. Population-based probability samples have an important role to play in understanding the population profiles accessing different STI services and continue to make a contribution to the efficient and clinically effective planning of comprehensive sexual health services, of which chlamydia testing is only a part.

**Key messages**

- The majority of chlamydia testing in Britain now occurs outside of genitourinary medicine (GUM), with those tested in general practice and other non-GUM settings less likely to report traditionally ascribed risk behaviours.
- However, a sizeable minority of those testing outside of GUM report higher-risk behaviours, of whom most have not attended GUM.
- Despite national recommendations that those diagnosed with chlamydia should be tested for other STIs including HIV, most individuals treated for chlamydia outside of GUM did not report an HIV test in the same time frame.
