# Supplementary material for: Patterns of chlamydia testing in different settings and implications for wider STI diagnosis and care: a probability sample survey of the British population
Source: Sex Transm Infect. 2016 Dec 15;93(4):276–83. doi: 10.1136/sextrans-2016-052719 (PMC5520351; doi:10.1136/sextrans-2016-052719)
Supplement: supplementary tables [file sextrans-2016-052719supp001.pdf]

Appendix table 1: Demographic characteristics of those tested for chlamydia in the past year, by location of most recent test (non-GUM and non-GP settings)

	NHS Family Planning Clinic		Antenatal service		School, college, university		Somewhere else*	
	%	(95% CI)	%	(95% CI)	%	(95% CI)	%	(95% CI)
**<u>Women</u>**								
Age(years)								
16-24	56.5	[47.3,65.3]	35.2	[25.0,47.0]	95.9	[90.6,98.2]	65.3	[56.5,73.2]
25-34	31.1	[23.4,40.1]	43.7	[32.3,55.9]	4.1	[1.8,9.4]	23.5	[17.4,30.9]
35-44	12.4	[6.4,22.7]	21.0	[10.8,37.0]	0.0	-	11.2	[5.8,20.5]
Country								
England	90.0	[84.3,93.8]	83.8	[73.8,90.5]	100.0	-	94.0	[88.5,96.9]
Scotland	5.3	[2.7,10.2]	9.3	[4.3,19.2]	0.0	-	3.9	[1.7,8.9]
Wales	4.7	[2.4,9.0]	6.9	[3.5,13.3]	0.0	-	2.1	[0.7,6.3]
Relationship status								
Live with a partner (incl married)	39.5	[30.4,49.2]	80.4	[71.2,87.1]	9.7	[5.1,17.5]	35.3	[27.5,44.1]
In a steady on-going relationship	33.4	[25.7,42.1]	9.6	[5.2,17.1]	49.2	[38.9,59.5]	32.7	[25.8,40.5]
Not in a steady relationship	27.1	[20.3,35.3]	10.0	[5.5,17.6]	41.2	[31.2,51.9]	32.0	[24.8,40.1]
Education								
No academic qualifications	8.4	[4.2,16.1]	11.0	[6.0,19.5]	0.0	-	8.1	[4.6,13.8]
Academic qualifications typically gained at age 16	36.9	[28.8,45.9]	38.6	[27.2,51.3]	8.8	[4.8,15.5]	28.0	[21.0,36.2]
Studying for/attained further academic qualifications	54.6	[45.4,63.5]	50.4	[38.5,62.2]	91.2	[84.5,95.2]	63.9	[55.6,71.6]
Student in full time education								
No	79.2	[71.2,85.5]	98.7	[91.6,99.8]	33.7	[24.2,44.8]	75.9	[67.7,82.6]
Yes	20.8	[14.5,28.8]	1.3	[0.2,8.4]	66.3	[55.2,75.8]	24.1	[17.4,32.3]
Area of residence								
Urban	87.8	[81.0,92.4]	80.8	[69.6,88.6]	80.4	[70.6,87.6]	85.4	[79.0,90.2]
Rural	12.2	[7.6,19.0]	19.2	[11.4,30.4]	19.6	[12.4,29.4]	14.6	[9.8,21.0]

	NHS Family Planning Clinic		Antenatal service		School, college, university		Somewhere else*	
	%	(95% CI)	%	(95% CI)	%	(95% CI)	%	(95% CI)
Denominator (unwt, wt)	*150, 86*		*81, 44*		*110, 65*		*173, 98*	
**Men**								
Age(years)								
16-24	(61.3)	[41.3,78.1]			95.5	[89.2,98.2]	73.7	[63.9,81.7]
25-34	(28.1)	[14.4,47.5]			3.5	[1.2,9.3]	21.0	[14.0,30.3]
35-44	(10.7)	[2.4,36.3]			1.0	[0.1,7.1]	5.2	[2.1,12.6]
Country								
England	(94.3)	[75.7,98.9]			99.4	[95.9,99.9]	93.4	[79.5,98.1]
Scotland	(4.6)	[0.6,26.1]			0.6	[0.1,4.1]	5.1	[1.1,21.1]
Wales	(1.2)	[0.2,7.9]			0.0	-	1.4	[0.3,6.2]
Relationship status								
Live with a partner (incl married)	(25.1)	[11.4,46.6]			4.0	[1.5,10.3]	19.3	[12.4,28.8]
In a steady on-going relationship	(27.6)	[14.2,46.8]			48.7	[38.6,58.9]	35.3	[26.1,45.7]
Not in a steady relationship	(47.3)	[29.4,66.0]			47.4	[37.5,57.4]	45.4	[35.9,55.3]
Education								
No academic qualifications	(15.0)	[6.0,33.1]			7.1	[1.9,23.4]	6.1	[3.1,11.6]
Academic qualifications typically gained at age 16	(29.5)	[14.9,50.0]			15.0	[9.4,23.1]	32.0	[23.7,41.6]
Studying for/attained further academic qualifications	(55.5)	[35.3,73.9]			77.8	[66.0,86.4]	61.9	[51.9,70.9]
Student in full time education								
No	(73.9)	[53.8,87.3]			41.4	[31.6,51.9]	67.1	[56.3,76.4]
Yes	(26.1)	[12.7,46.2]			58.6	[48.1,68.4]	32.9	[23.6,43.7]
Area of residence								
Urban	(77.7)	[59.4,89.2]			77.5	[68.1,84.7]	92.9	[87.6,96.0]
Rural	(22.3)	[10.8,40.6]			22.5	[15.3,31.9]	7.1	[4.0,12.4]
Denominator (unwt, wt)	*32, 24*		*0, 0*		*127, 89*		*140, 112*	

*Excludes GUM and GP; Denominator is those aged 16-44 reporting a chlamydia test in the last year, and most recent test was at a setting other than GUM or GP; ( ) indicates small denominators (n<50)

Appendix table 2: Behavioural characteristics of those tested for chlamydia in the past year, by location of most recent test (non-GUM and non-GP settings)

	NHS Family Planning Clinic		Antenatal service		School, college, university		Somewhere else*	
	%	(95% CI)	%	(95% CI)	%	(95% CI)	%	(95% CI)
**Women**								
Number of partners[a], past year								
1	63.0	[53.7,71.4]	90.2	[81.2,95.2]	57.2	[46.6,67.2]	55.6	[47.1,63.8]
2	15.9	[10.3,23.6]	2.1	[0.7,6.3]	22.5	[15.0,32.2]	19.2	[13.5,26.6]
3+	21.1	[14.7,29.4]	7.7	[3.3,16.9]	20.3	[13.4,29.5]	25.2	[18.5,33.2]
Same-sex partner(s), past year	2.9	[1.0,7.8]	1.5	[0.2,9.9]	4.5	[1.6,12.0]	5.1	[2.8,9.3]
Unsafe sex, past year[b]	11.1	[6.9,17.4]	5.5	[2.1,13.7]	12.3	[7.2,20.2]	17.8	[12.2,25.2]
Concurrency in past year[c]	12.2	[7.1,20.2]	5.5	[1.9,15.1]	18.5	[11.2,29.1]	18.7	[12.7,26.8]
New partners from outside UK, past 5 years[d]	13.2	[8.1,20.9]	8.8	[3.8,19.0]	3.6	[1.3,9.9]	12.9	[8.1,20.0]
A sexual partner was concurrent, past 5 years (yes/probably)	51.4	[41.8,60.8]	41.5	[30.1,53.9]	44.5	[34.0,55.5]	51.5	[43.0,60.0]
Denominator (unwt, wt	*150, 86*		*81, 44*		*110, 65*		*173, 98*	
**Men**								
Number of partners[a], past year								
1	(55.5)	[36.8,72.8]			52.3	[42.0,62.3]	46.5	[36.6,56.7]
2	(15.8)	[6.2,34.5]			21.9	[15.2,30.4]	29.8	[20.5,41.1]
3+	(28.7)	[14.8,48.2]			25.9	[18.3,35.2]	23.7	[16.9,32.1]
Same-sex partner(s), past year	(2.3)	[0.3,14.7]			5.4	[2.6,11.1]	1.4	[0.5,3.9]
Unsafe sex, past year[b]	(11.1)	[4.1,26.8]			6.5	[3.2,12.7]	11.4	[6.7,18.5]
Concurrency in past year[c]	(15.5)	[5.5,36.8]			17.5	[11.2,26.4]	21.2	[12.4,34.0]
New partners from outside UK, past 5 years[d]	(19.0)	[8.5%,37.1%]			14.4 %	[8.8,22.7]	13.4	[8.2,21.3]
A sexual partner was concurrent, past 5 years (yes/probably)	(44.5)	[27.2,63.3]			39.0	[29.4,49.4]	51.8	[41.6,61.9]
Denominator (unwt, wt)	*32, 24*		*0, 0*		*127, 89*		*140, 112*	

[a]opposite- and/or same-sex partners; [b]defined as two or more partners in the past year, and not used a condom in the past year; [c]Overlap between any of three most recent partners in the past 5 years; [d]includes partners acquired when abroad and when in the UK; *Excludes GUM and GP; Denominator is those aged 16-44 reporting a chlamydia test in the last year, and most recent test was at a setting other than GUM or GP; [ ] indicates small denominators (<50);

Appendix table 3: HIV testing (past 5 years) and reasons for having an HIV test by location of chlamydia treatment in the past year

	Women				Men			
	GUM		GP/other settings[b]		GUM		GP/other settings[b]	
	%	[95% CI]	%	[95% CI]	%	[95% CI]	%	[95% CI]
HIV test (past 5 years)	74.1	[65.9,80.8]	54.5	[42.7,65.7]	65.8	[56.2,74.3]	23.9	[12.7,40.5]
Denominator (unwt, wt)	*162, 81*		*99, 56*		*125, 106*		*38, 33*	
Reasons for HIV testing[a]:								
Pregnancy	20.0	[13.3,29.0]	29.9	[18.1,45.1]	1.9	[0.5,7.5]	[-]	[-]
Travel/insurance/ mortgage	1.9	[0.3,12.3]	0.0	-	0.0	-	[-]	[-]
Sexual health check	65.6	[53.9,75.6]	53.9	[38.1,68.9]	77.1	[66.0,85.3]	[-]	[-]
General health check	13.2	[6.1,26.4]	11.6	[4.8,25.3]	15.5	[8.7,26.0]	[-]	[-]
Stopped using condoms	0.5	[0.1,3.5]	1.9	[0.5,7.5]	4.1	[1.3,12.6]	[-]	[-]
Concern of risk of self/partner	4.9	[1.4,10.7]	11.0	[2.5,37.1]	6.7	[2.7,15.9]	[-]	[-]
Doctor's advice	2.9	[1.1,7.9]	1.0	[0.1,6.7]	4.2	[1.0,15.9]	[-]	[-]
Other reasons	2.8	[1.0,7.8]	9.2	[3.3,23.2]	1.6	[0.4,6.9]	[-]	[-]
Denominator (unwt, wt)	*205, 122*		*79, 48*		*114, 96*		*22, 18*	

Denominator is those aged 16-44 who reported being treated for chlamydia in the past year. Denominator for reasons is further restricted to those with an HIV test in the past 5 years; [a]Participants could give more than one reason therefore columns sum to more than 100%; Reasons do not necessarily relate to most recent HIV test if participants has tested more than once; [b]combined due to small denominators; [-] Results not presented due to small denominators (n<30)
